# The association between oxidative stress and endothelial dysfunction in early childhood patients with Kawasaki disease

**DOI:** 10.1186/s12872-018-0765-9

**Published:** 2018-02-09

**Authors:** Takamichi Ishikawa, Keigo Seki

**Affiliations:** Department of Pediatrics, Hamamatsu University School of Medicine, 1-20-1 Handayama, Higashi-ku, Hamamatsu, 431-3192 Japan

**Keywords:** Oxidative stress, Kawasaki disease, Endothelial dysfunction, Total fever duration, Children

## Abstract

**Background:**

Oxidative stress has recently been shown to play an important role in the development of arteriosclerosis in patients with Kawasaki disease (KD); however, no study has investigated this association in early childhood patients with KD. In this study, we evaluated prospectively the association between the levels of oxidative stress and the endothelial function in early childhood patients with KD.

**Methods:**

We compared the derivatives of reactive oxygen metabolites (ROM), flow-mediated dilatation (FMD), and biological characteristics in a population of 50 children: 10 patients with KD and coronary artery lesions (CAL) (group 1), 15 KD patients without CAL (group 2), and 25 healthy age- and sex-matched children (group 3).

**Results:**

The median age of all KD children at study enrollment was 6.8 (IQR 4.4–8.2) years. ROM levels were significantly higher in group 1 (*p* <  0.001) and group 2 (*p* = 0.004) than in group 3. The %FMD of group 1 (*p* <  0.001) and group 2 (*p* = 0.026) was significantly lower than that of group 3. There was a significant negative correlation between ROM and %FMD (r = − 0.60, *p* <  0.001). A multiple linear regression analysis identified ln-ROM (standardized coefficient = − 0.403, *p* = 0.043) and total fever duration (standardized coefficient = − 0.413, *p* = 0.038) as significant determinants of %FMD in the patients with KD.

**Conclusions:**

Our study suggests that oxidative stress is strongly associated with endothelial dysfunction in early childhood patients with KD. Furthermore, we found that the longer the fever duration, the higher the risk of oxidative stress-induced endothelial dysfunction in these children.

## Background

Kawasaki disease (KD) is characterized by systemic vasculitis, and occurs most frequently in infants and young children. A history of KD has recently been indicated as a possible risk factor for the early onset of arteriosclerosis. Endothelial dysfunction is one of the earliest manifestations of arteriosclerosis [1, 2] and has been demonstrated in KD children with or without coronary artery lesions (CAL) after acute illness [3, 4]. While these mounting evidences suggest that there is premature arteriosclerosis in patients with a history of KD, the underlying mechanisms are still undefined.

Oxidative stress is caused by the presence of reactive oxygen species (ROS). Excessive ROS production represents endothelial and smooth muscle dysfunction, which leads to the progression of atherosclerosis [5, 6]. Recently, the derivatives of reactive oxygen metabolites (ROM) test, which mainly consists of measuring hydroperoxide levels as a marker of ROS, has been used to directly assay the total oxidant capacity [7–9].

It has been increasingly recognized that oxidative stress plays an integral role in the development of arterial endothelial dysfunction and the risk of premature arteriosclerosis in KD patients. However, most of the previously reported studies investigated the association between oxidative stress and the endothelial function in adolescents and adults with KD late after the onset of the disease [10–12]. In the present study, we evaluated the association between the levels of oxidative stress and the endothelial function in early childhood patients with KD. In addition, we determined whether any differences existed in the factors associated with oxidative stress and the endothelial function in these children.

## Methods

We enrolled two groups of KD patients with and without CAL and a group of age- and sex-matched healthy controls (1:1 matching). All of the patients with KD were diagnosed in or referred to the Hamamatsu University Hospital in Japan. The KD patients who met the following criteria were recruited: (1) a diagnosis of KD that was established by the Japanese Kawasaki Disease Research Committee, and (2) an interval from the initial onset of illness of up to five years. We obtained the data for all subjects from their clinical records. As a control group, we recruited healthy children who had undergone a health checkup with no evidence of disease history and with a normal physical examination. Controls matched according to sex and date of birth (within 1 year) were selected from these children. The subjects were recruited during the study period between April 2015 and September 2016. The institutional Ethics Committee approved the study and the parents of all of the participants provided their written informed consent.

We classified the participants into three groups: group 1 comprised the patients with CAL, whether persistent or regressed, group 2 comprised the patients without CAL at any time, and group 3 comprised age- and sex-matched healthy control children. The coronary artery segments measured by echocardiography included the internal lumen diameters of the left main coronary artery, left anterior descending artery, and right coronary artery. The coronary artery dimensions were normalized for body surface area as z-scores and calculated by regression eqs. [13]. Patients were considered to have CAL if any coronary artery segment had a z-score ≥ 2.5 and were excluded to have CAL if any coronary artery segment had a z-score < 2.5.

Fasting total cholesterol (TC), high-density lipoprotein cholesterol (HDL-C), low-density lipoprotein cholesterol (LDL-C), and triglyceride levels were determined. The plasma TC, HDL-C, triglyceride, and uric acid levels were determined by conventional enzymatic methods. The concentration of LDL-C was calculated by the Friedewald formula. The glycosylated hemoglobin A1c was measured by electrophoresis. The plasma N-terminal-pro-B-type natriuretic peptide (NT-proBNP) concentration was measured on an Elecsys2010 analyzer with a chemiluminescent immunoassay kit (Roche Diagnostics GmbH, Mannheim, Germany). We also calculated the TC/HDL-C ratio by dividing TC by HDL-C. The C-reactive protein (CRP) level was measured with a highly sensitive assay, which is a particle-enhanced immunoturbidimetric assay consisting of an anti-CRP monoclonal antibody coupled to latex microparticles (Roche Diagnostics). The assay is standardized against a CRM 470 reference preparation for proteins in human serum and has a functional sensitivity of 0.1 mg/L.

The principle of the ROM test has been described previously [7, 8]. We measured hydroperoxide levels as serum ROM levels in children in the present study using a Free Radical Elective Evaluator (FREE®; Wismerll Co. Ltd., Tokyo, Japan). The ROM test spectrophotometrically detects the oxidization of N,N-diethyl-para-phenylenediamine as a chromogenic substrate by radicals converted from hydroperoxide. To measure ROM, a 20-μL serum sample and 1 mL of buffered solution (R2 kit reagent) were gently mixed in a cuvette, and 20 μL of chromogenic substrate (R1 kit reagent) was then added to the cuvette. After mixing well, the cuvette was immediately incubated in the thermostatic block of the analyzer for 5 min at 37 °C and the absorbance at 505 nm was recorded [14]. Measurements are expressed as an arbitrary unit called the Carratelli unit (U.CARR) such that 1 U.CARR corresponds to 0.8 mg/L H_2_O_2_. The normal reference level of ROM was 250 to 300 U.CARR [7, 8].

Endothelial function was measured using flow-mediated dilatation (FMD) performed by a single trained researcher (T.I.) blinded to the subject’s status by a randomization code, according to the guidelines and standards [15, 16]. The diameter of the brachial artery was measured on high-resolution, two-dimensional ultrasound images obtained by Philips HD11XE (Philips Medical Systems, Andover, MA, USA) devices with a 12 L linear array transducer. A sphygmomanometer cuff used for vascular occlusion was positioned distal to the ultrasound probe and inflated to a pressure of 200 mmHg for 5 min, then deflated suddenly to induce an increased blood flow. Brachial artery diameter was measured from the anterior to the posterior interface between the media and adventitia. The mean diameter of the brachial artery was calculated from three cardiac cycles gated with an electrocardiogram. The peak percent FMD (%FMD) was defined as the percentage change relative to that of the initial resting scan.

A 10-min rest was provided after the FMD scan for vessel recovery, and the non-endothelial-dependent brachial artery dilatation was assessed with two-dimensional imaging before and 3 to 4 min after the administration of sublingual glyceryl trinitrate (GTN) spray (0.3 mg). The peak %GTN was defined as the percentage change in the arterial diameter after the administration of GTN relative to the baseline vessel diameter.

The same examiner performed the measurements of the intima-media thickness (IMT), FMD, and GTN throughout the study. The IMT of the common carotid artery was measured at high resolution with B-mode ultrasound images obtained by the same devices and transducer. For the measurements of IMT, longitudinal images of the carotid arteries were obtained [17]. The distance between the leading edges of the lumen-intima interface and the media-adventitia interface of the B-mode frame was considered. A software program (Qlab; Philips Medical Systems) was used to analyze the IMT distance automatically at 64 points within a 10-mm segment. The value given was the calculated arithmetic mean IMT.

Data are presented as the mean ± standard deviation and median (interquartile range: IQR) as appropriate. Data normality was initially verified using the Shapiro-Wilk test allowing the use of parametric statistical analysis. To compare the differences between the groups, a one-way analysis of variance (ANOVA) and post hoc Tukey’s test were used for the parametric variables, the Kruskal-Wallis test with post hoc comparison by Dunn’s multiple comparison test for the non-parametric variables, and Fisher’s exact test for the categorical variables. When a significant difference was indicated by ANOVA, the specific source of the difference was identified using either the Mann-Whitney U test or paired t-test, with Bonferroni correction. For comparison of non-parametric variables between two groups, the Mann-Whitney U test was used. The correlations were analyzed using Spearman’s correlation coefficient by rank. Because the ROM levels were not normally distributed, we calculated the natural logarithmic transformed ROM as ln-ROM to be used for regression analysis. A multiple regression analysis was performed in KD patients to examine the associations between %FMD and the clinical parameters, including the number of days with a fever before diagnosis, total fever duration, white blood cell (WBC) count, CRP level, age, sex, body mass index (BMI), and ln-ROM after a univariate analysis. For this purpose, a linear regression model was used in a stepwise method with a probability of 0.05 to enter and 0.10 to remove. Significance was defined as a *p* value of less than 0.05.

## Results

A total of 50 children were studied. Group 1 comprised 10 patients, six of whom had persistent coronary aneurysms and four of whom had regressed aneurysms. None had symptoms of myocardial ischemia and none required coronary artery interventions. Group 2 comprised 15 patients with KD and excluded CAL. All of the 25 patients with KD had received intravenous immunoglobulin (IVIG) during the acute phase of illness, and six were continuing to take long-term, oral low-dose aspirin. Of the 25 KD patients, 18 children completely responded to a single IVIG therapeutic session, and seven patients required a second dose of IVIG because of a persistent fever; however, of these seven patients, six failed to respond to the second IVIG therapy session. In these six children, one patient received plasma exchange therapy, four patients received prednisolone therapy and one patient required infliximab therapy. We performed coronary angiography (CAG) in all ten patients in Group 1, not in the remaining 15 children without CAL. The median age of all KD children (groups 1 and 2) at study enrollment was 6.8 years (IQR 4.4–8.2 years). The patients with KD were studied at a median time interval of 3.9 years (IQR 1.4–4.8 years) from the onset of disease. Group 3 comprised 25 age- and sex-matched healthy children.

The baseline and biological characteristics of all patients are shown in Table 1. There was no significant difference in any of the parameters among the three groups. The levels of TC, HDL-C, LDL-C, TC/HDL-C ratio, triglyceride, uric acid, hemoglobin A1c, and NT-proBNP were all within normal limits and were not significantly different among the three groups.

**Table 1:** Clinical and biological characteristics of the study group

	Group 1 (*n* = 10)	Group 2 (*n* = 15)	Group 3 (*n* = 25)	p Value
Age (years)	7.5* (4.3–11.7)	6.7* (5.0–7.6)	6.4* (5.8–9.4)	0.611
Sex (male/female)	4/6	8/7	12/13	0.832
Age at diagnosis of KD (years)	2.9* (1.2-6.4)	2.1* (1.0-3.2)	NA	0.952
Follow-up interval (years)	3.4* (1.1-3.9)	4.5* (4.0-4.9)	NA	0.243
Height (cm)	122.0 ± 29.2	111.8 ± 15.5	119.7 ± 15.7	0.450
Weight (kg)	28.4 ± 13.6	21.0 ± 6.3	23.0 ± 7.5	0.514
BMI (kg/m2)	17.2 ± 2.3	16.5 ± 1.6	15.7 ± 1.5	0.120
Systolic BP (mm Hg)	94.4 ± 5.7	95.8 ± 6.0	96.4 ± 7.0	0.807
Diastolic BP (mm Hg)	54.0 ± 5.4	53.6 ± 6.9	55.7 ± 5.2	0.234
HR (beats/min)	87.0 ± 17.4	91.9 ± 17.8	86.5 ± 13.5	0.324
TC (mg/dL)	183.6 ± 30.6	168.9 ± 21.6	170.6 ± 22.7	0.247
HDL-C (mg/dL)	61.0 ± 26.0	58.8 ± 11.5	62.8 ± 12.2	0.335
LDL-C (mg/dL)	97.9 ± 13.1	92.3 ± 15.5	90.6 ± 24.3	0.593
TC/HDL-C	3.3 ± 0.8	3.0 ± 0.6	2.8 ± 0.6	0.458
Triglyceride (mg/dL)	94.3 ± 47.1	88.3 ± 37.7	79.4 ± 39.5	0.483
Uric Acid (mg/dL)	4.5 ± 0.9	3.9 ± 0.8	3.9 ± 1.2	0.180
Hemoglobin A1c (%)	5.2 ± 0.4	5.4 ± 0.3	5.4 ± 0.3	0.869
NT-pro BNP (pg/mL)	42.8 ± 19.7	61.2 ± 24.8	55.5 ± 21.8	0.192

*The values are presented as the median (interquartile range)

**Fig. 1:**
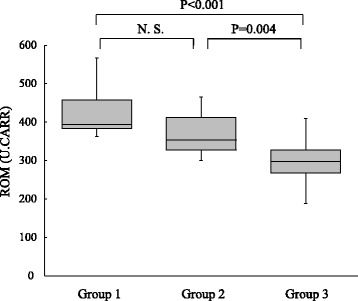
Box plots showing the distribution of ROM in three groups. The upper boundary of the box represents the 75th percentile, and the lower boundary of the box represents the 25th percentile. The line through each box represents the median value in each group. ROM, derivatives of reactive oxygen metabolites

The ROM levels differed significantly among the three groups, being significantly higher in group 1 (median 394 U.CARR, IQR 383–458 U.CARR) than in group 3 (median 298 U.CARR, IQR 268–327 U.CARR, *p* <  0.001). The ROM levels in group 2 (median 353 U.CARR, IQR 328–412 U.CARR) were also significantly greater than in group 3 (*p* = 0.004) (Fig. 1).

**Fig. 2:**
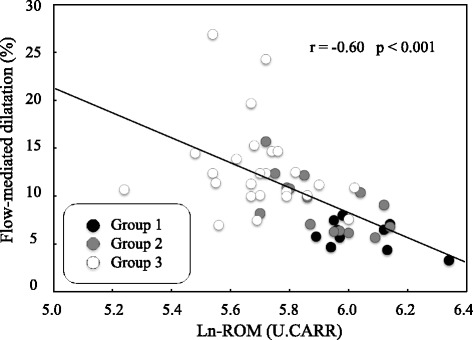
A scatter plot and correlation between %FMD and ROM. FMD, flow-mediated dilatation; ROM, derivatives of reactive oxygen metabolites

The %FMD was significantly lower in group 1 (6.0 ± 1.5%) than in group 3 (12.9 ± 4.7%, p <  0.001). The %FMD was also reduced significantly in group 2 (9.2 ± 2.9%) in comparison to group 3 (*p* = 0.026). There were no significant differences in %GTN (*p* = 0.611) and IMT (*p* = 0.425) between the three groups (Table 2). The intraobserver variability in the measurements of the FMD, GTN and IMT was 6.8%, 5.4%, and 4.6%, respectively. Importantly, there was a significant negative correlation between ROM and %FMD (r = − 0.60, *p* <  0.001) (Fig. 2).

**Table 2:** Comparison of FMD, GTN-mediated dilatation, and Carotid IMT in the three groups

	Group 1 (n = 10)	Group 2 (n = 15)	Group 3 (n = 25)	p Value (ANOVA)	p Value (post hoc test)		
					1 vs. 3	2 vs. 3	1 vs. 2
FMD (%)	6.0 ± 1.5	9.2 ± 2.9	12.9 ± 4.7	< 0.001	< 0.001	0.026	0.082
GTN-mediated dilatation (%)	22.4 ± 6.2	25.0 ± 5.0	25.3 ± 5.0	0.611			
Carotid IMT (mm)	0.44 ± 0.03	0.44 ± 0.03	0.42 ± 0.04	0.425

In the patients with KD, the number of days with a fever before diagnosis was greater and the total fever duration was significantly longer in group 1 than in group 2 (*p* = 0.003 and *p* < 0.001, respectively). The serum CRP concentration in group 1 was greater than that in group 2 (*p* = 0.001). However, there was no significant difference in the WBC count between the two groups (*p* = 0.268) (Table 3). We also investigated the correlations between %FMD and these parameters added to age, sex, BMI, and ln-ROM in patients with KD. Using a univariate analysis, we found that the number of days with a fever before diagnosis, total fever duration, CRP levels, and ln-ROM inversely correlated with %FMD. To establish the independent predictors of FMD in patients with KD, a stepwise multiple linear regression analysis including these variables was performed; ln-ROM (standardized coefficient = − 0.403, *p* = 0.043) and total fever duration (standardized coefficient = − 0.413, *p* = 0.038) were independently associated with %FMD (Table 4).

**Table 3:** Clinical characteristics of patients with and without coronary involvement

	Group 1 (*n* = 10)	Group 2 (*n* = 15)	p Value
Days of fever before diagnosis	6.4 ± 1.8	4.6 ± 0.9	0.003
Total fever duration (days)	10.5 ± 2.5	5.5 ± 1.6	< 0.001
WBC (cells/μL)	16300* (12525–19,800)	14600* (12250–16,150)	0.268
CRP (mg/dL)	14.6* (10.9–19.2)	7.6* (5.2–8.1)	0.001

*The values are presented as the median (interquartile range)

**Table 4:** Univariate analysis and multiple regression analysis of the relation between %FMD and variables in patients with KD

	Univariate analysis		Multiple regression analysis	
	Regression coefficient	p Value	Standardized coefficient (95% CI)	p Value
Age (year)	0.027	0.449		
Sex	0.261	0.103		
BMI (kg/m^2^)	−0.173	0.204		
Days of fever before diagnosis	−0.489	0.007		
Total fever duration (days)	−0.671	< 0.001	- 0.413 (−0.732 to − 0.022)	0.038
WBC (cells/μL)	−0.167	0.213		
CRP (mg/dL)	−0.517	0.004		
Ln-ROM (U. CARR)	−0.667	< 0.001	- 0.403 (−14.741 to −0.274)	0.043

## Discussion

The present study showed the following: (1) a significant increase in levels of ROM, an oxidative stress marker, in early childhood patients after KD; (2) a significant negative correlation between ROM and %FMD in the study population; and (3) that ROM and total fever duration were closely associated with %FMD in the KD children.

Our study population comprised younger patients whose median age and elapsed time from the initial onset were 6.8 and 3.9 years, respectively. By contrast, the study populations in most previously reported studies that assessed oxidative stress and endothelial function comprised teenage or adult patients [10–12]. This is the first report to investigate the association between oxidative stress and the endothelial function in early childhood patients with KD.

The ROM levels in the acute stage of KD have recently been described by Yahata et al. [18]. Although ROM levels were reduced by IVIG therapy in these patients, the levels at two weeks after the IVIG treatment (430 U.CARR) remained higher than the normal range. However, the state of oxidative stress between the acute and late phase of KD was unclear. The results of the present study confirmed that the ROM levels in KD patients with and without CAL were significantly greater than in normal controls, and suggest that oxidative stress is persistent after the acute phase of KD.

We demonstrated in this early childhood study that there was a significant negative correlation between ROM and %FMD, which has been widely regarded as a marker of the endothelial function. It is well known that the grade of the endothelial function is a predictor of cardiovascular outcomes in adults. Higashi et al. demonstrated the role of oxidative stress in the endothelial dysfunction. They described one of the possible mechanisms whereby reduced nitric oxide (NO) and increased oxidative stress impaired the endothelium-dependent vasodilation [19]. Accumulating evidence suggests that endothelial dysfunction may be caused by the accelerated inactivation of nitric oxide by ROS, which reduces the bioavailability of NO [20]. Our findings of a correlation between ROM and %FMD strongly support these speculations. Based on our results, ROM, which reflects oxidative stress, may be a useful biomarker to evaluate the presence of endothelial dysfunction in children with KD.

Endothelial dysfunction is an early feature of both arteriosclerosis and vascular diseases in humans [1, 2]. In addition, endothelial dysfunction is an earlier manifestation of arteriosclerosis than carotid IMT thickening. In the present study, there was no significant difference in the IMT between the KD patients and the controls. Carotid IMT thickening is postulated to be secondary to the changes in the arterial walls after diffuse vasculitis due to KD [21]. It is conceivable that IMT thickening was not detected in this study because our study population was younger than that in previous studies [22, 23]. In young children, especially those within five years of the onset of KD, FMD impairment occurs prior to intima-media complex thickening. Our results suggest that oxidative stress may cause functional changes in the arterial wall in the premature arteriosclerosis earlier than the anatomical changes in KD patients within five years after the onset of illness.

In this study, among KD patients with CAL, the inflammatory process was more severe than that observed in the remaining children (greater number of days with a fever before diagnosis and the total fever duration and increased CRP levels). Dalla et al. also reported that the patients with coronary arterial involvement had increased CRP levels at the time of presentation of the illness [24]. The American Heart Association and American Academy of Pediatrics guidelines recommend that IVIG be administered to children with KD within the first 10 days of illness, and if possible, within the first seven days of illness [21]. Coronary artery aneurysms occur significantly more often in patients with a delayed diagnosis of KD, especially when the total duration of fever is longer than eight days [25, 26]. Importantly, the current study documented that ROM and total fever duration were independently associated with %FMD in the patients with KD. These results suggest that a prolonged inflammatory process causes not only coronary artery aneurysm but also systemic endothelial dysfunction caused by persistent oxidative stress. The association between increased oxidative stress and subclinical inflammation amplifies the signals that lead to vascular damage [27, 28]. Proinflammatory cytokines and advanced glycation end-products generate the intracellular ROS responsible for the activation of redox-sensitive transcription factors and phenotypical changes in endothelial cells, including the expression of adhesion molecules and tissue factor and the release of proinflammatory cytokines that characterize endothelial dysfunction [29]. Our study showed that a longer duration of fever is associated a higher risk of oxidative stress-induced endothelial dysfunction. We believe that the duration of fever is as important with CAL as without CAL in regard to endothelial dysfunction and the genesis of arteriosclerosis. For this reason, the patients with prolonged fever during KD should take care regarding arteriosclerosis, even if they have no CAL. Therefore, shortening the total fever duration is important for the prevention of early arteriosclerosis in the KD patients.

This study has some limitations. First, it was a small study of only 25 children with KD and 25 controls, so the findings should be considered as preliminary. Larger studies are needed to confirm the differences in oxidative stress and the endothelial function between control children and the patients with KD. Second, the fact that patients were not receiving the same treatment may affect the results of the present study. In particular, additional treatment such as steroid and infliximab and plasma exchange may have influenced the results. Third, we performed CAG to only Group 1 children. We may have been able to demonstrate coronary arteriosclerosis or atherosclerosis in more detail if all patients with KD had received CAG. Fourth, we did not assess the levels of oxidative stress and the endothelial function in the different phases of KD. Further investigations with a long follow-up period are needed to clarify the true risk for arteriosclerosis in patients with KD.

## Conclusions

The present study evaluated the oxidative stress and the endothelial function after KD in the youngest patients reported to date. In conclusion, our study suggests that oxidative stress may have a pathogenic role in the functional changes of the arterial wall even in early childhood, especially within five years of the onset of KD. In addition, we found that a longer duration of fever results in a higher risk of endothelial dysfunction caused by persistent oxidative stress. Patients with a persistent or recurrent fever during KD, such as non-responders to IVIG, may require long-term follow-up to monitor the genesis of arteriosclerosis.

## Abbreviations

ANOVA: Analysis of variance
BMI: Body mass index
CAL: Coronary artery lesions
CRP: C-reactive protein
ECG: Electrocardiogram
FMD: Flow-mediated dilatation
GTN: Glyceryl trinitrate
HDL-C: High-density lipoprotein cholesterol
IMT: Intima-media thickness
IQR: Interquartile range
IVIG: Intravenous immunoglobulin
KD: Kawasaki disease
LDL-C: Low-density lipoprotein cholesterol
NO: Nitric oxide
NT-proBNP: N-terminal-pro-B-type natriuretic peptide
ROM: Reactive oxygen metabolites
ROS: Reactive oxygen species
SD: Standard deviation
TC: Fasting total cholesterol
U.CARR: Carratelli unit
WBC: White blood cell
